# Medical Journals and Medical Schools

**Published:** 1837

**Authors:** 


### Art. VII—Medical Journals and Medical Schools.

The founders of the Medical Department of the Cincinnati College have, by *certain* persons, been seriously blamed, from the hour when they commenced the work, for attempting to add another school to that already in the city. It was said, "*the Medical College of Ohio, and Transylvania University, are sufficient for the West; even for years to come, they can receive and educate all our students; and you will do harm to the profession by starting another.*" We are not about to answer these assertions, but have quoted them for a very different purpose. If they are valid when applied to a medical school, they must be still more so, when applied to a medical journal; for more schools than journals can be supported in every country. But have they been thus applied? By no means. In the year 1827, the writer of this article began the Western Journal, when no other existed in the West. He belonged to no medical school, and directed his periodical against none. Soon *afterwards*, however, the Transylvania professors put forth a similar work. Of this he never thought of complaining. In the year 1833, certain professors of the Medical College of Ohio, commenced another, of which still he did not complain. It fell into a state of suspended animation, was resuscitated, and then expired. In 1835, it was succeded by a third, under their immediate patronage, which lived not half a year. In 1837, they brought out a fourth, but soon left it to perish. Of all *this* he did not complain. But the very persons, who did all this, were they, who labored to stir up the community against the founders of the Cincinnati College. We shall quote upon them their own argument, substituting the word Journal for school: "*The Western Journal, and the Transylvania Journal, are sufficient for the West; even for years to come, they can supply all the wants of our physicians; and you will do harm to the profession by starting another.*"

With this presentation of their inconsistency, we shall leave them to their own, and the *reflections* of a candid community.

